# The Potential of Digital Impression in Orthodontics

**DOI:** 10.3390/dj10080147

**Published:** 2022-08-08

**Authors:** Sabina Saccomanno, Stefano Saran, Valeria Vanella, Rodolfo Francesco Mastrapasqua, Luca Raffaelli, Luca Levrini

**Affiliations:** 1 Department of Health, Life and Environmental Science, University of L’Aquila, Piazza Salvatore Tommasi, 67100 L’Aquila, Italy; 2 Department of Human Sciences, Innovation and Territory, School of Dentistry, Postgraduate of Orthodontics, University of Insubria, 21100 Varese, Italy; 3 Dental School, Catholic University of the Sacred Heart, 00168 Rome, Italy; 4 ENT Department, Rivoli Hospital, ASL TORINO 3, 10098 Torino, Italy

**Keywords:** dental impression technique, dental impression materials, technology, dental, diagnosis, oral, digital work-flow, aligners

## Abstract

Background: Over the past 20 years, there have been many innovations in orthodontic diagnosis and therapy. Among the innovations, there is the taking of dental impressions (DIs). Dental impressions are the negative imprint of hard and soft tissues of one or both arches, and they allow a plaster model to be formed, i.e., a positive reproduction. Traditional dental impressions can be made of different materials, such as alginate, while digital impression is captured by an intra-oral scanner. Digital impression, despite the evident advantages, has not yet replaced the conventional impression. The aim of this study is to evaluate which dental impressions are the most used by dentists. For this purpose, we considered 120 questionnaires sent electronically to patients of different dental private practices from different countries, where the dentists can use both techniques. The results highlighted that the kind of impression adopted is very much influenced by the type of therapy and orthodontic devices used in the treatment. We can conclude that, despite the advent of digital technology, conventional impressions are still used for fixed devices, while digital impressions are more adopted for orthodontic customized devices and therapies with clear aligners, that are very widespread among adult patients.

## 1. Introduction

Conventional impression techniques allow an imprint of dental arches to be obtained to form a plaster model. Depending on the impression to be taken, traditional techniques involve the use of one or more impression materials with metal or plastic trays. Clinicians can use different materials with different properties: some materials, such as polyethers and polyvinyl siloxanes, can reproduce the anatomical details of the oral cavity more precisely than others. In the conventional impression method, the tray is filled with a soft paste and inserted into the patient’s mouth, where it is then held in that position until the paste itself has completely hardened, which takes a few minutes [1].

Nowadays, thanks to the improvement in dental materials and the advent of technology, new techniques, such as digital impression, have also been developed.

The digital dental impression (DI) occurs thanks to the new scanner technology. The intraoral scanner is a three-dimensional (3D) device capable of detecting dental impressions, first through the acquisition of a large number of images and, then, subsequent processing using dedicated software [2,3]. One of the most obvious advantages of using this technology is the drastic reduction in discomfort for patients, usually reluctant to take impressions with traditional methods (impression trays filled by alginate, silicone or polyether). In the orthodontic field, the use of digital flow and digital impressions is certainly very widespread, in part due to the fact that orthodontics is very common in children (generally less cooperative than adult patients), and in part because some orthodontic devices, such as aligners, if produced from digital impressions, allow the dentist to save a lot of time and to reduce the costs [4]. The taking of impressions with conventional techniques represents for many patients a very stressful step of dental therapy, and so, generally, the possibility of taking digital impression is appreciated [5,6].

Digital dental impression is detected thanks to the use of scanners. Scanners are digital intraoral imaging devices and consist of data acquisition systems of the tooth surface in three dimensions. They enable an almost completely digital workflow and, in many cases, have replaced the need to take impressions with alginate and then develop the plaster models [7]. All the various intraoral digital scanning devices are based on optical principles such as blue light emitting diodes (LEDs) and blue laser technology. The system consists of the detection of several single images processed together and in a continuous acquisition (streaming) of optical images [2]. Another important aspect is the accuracy of the impression taken. Accuracy is defined by two methods: trueness and precision. Trueness indicates the nearness of agreement between the mathematical mean of a great number of test results and the true or accepted reference value. Precision refers to the closeness of agreement between test results. The measurement method contributes to the variability of trueness and precision linked to the intraoral scanner (IOS), as this depends on variables such as the operator, equipment used and calibration, the time elapsed between measurements and the environment (temperature, humidity, etc.). However, the methods to calculate precision and trueness for IOS are limited due to the quality of references used and the measurement technique employed [8]. According to recent studies that tried to make a comparison between the accuracy of models produced by digital scanners and those produced by alginate or silicone, it appears that the digital scanner is much more precise. Tomita et al. measured the impressions taken by silicone, alginate and intraoral scanners. When they compared five lines measurements, they affirmed that intraoral scanners appear more accurate [9]. Another study compared the accuracy in the vertical preparation in prosthodontics; the results demonstrated the great potential of intraoral scanners [10]. The employment of IOS to perform the vertical preparation digitally for dental supported prosthesis with reasonable values determines no relevant differences in comparison with conventional impression [10]. However, from other studies and reviews of the literature, it is quite clear that accuracy of digital scanners will and should improve in the next several years, and there is an important need for other research about the precision of these devices [11,12]. In addition, digital impression also yielded a high inter-operator reproducibility: an in vivo study, carried out by Kamimura et al., showed that inter-operator reproducibility with DI is better than with conventional methods and, according to this study, this advantage of DI was not dependent on the clinical experience of the operator or on the oral condition of the patient. The region of the arches with the largest discrepancy was the lingual distal surface of the second molar, but only with a traditional impression. This is a problematic area to manage due to the accumulation of saliva and the difficulty of covering it completely with the tray. Another important aspect to consider is that digital impression is not subject to the typical dimensional change of many impression materials that can lead to inaccuracies [13]. It is important, especially in prosthetic therapies, because an imperfect marginal adaptation can compromise the treatment. Despite the technological advent, digital technology for impressions has not yet become a routine in the practice of all dentists and laboratories. For this reason, especially in the orthodontic field, the use of techniques such as aligners has been limited compared to the rest of orthodontics. Unfortunately, the main limitation of the intraoral scanner is still its high cost, which limits its use to a limited number of operators and laboratories. Moreover, operators need some time to learn the technology before becoming proficient, and this, together with costs, has prevented the spread of the method [14,15].

The purpose of this study is to evaluate how much digital impressions are used in orthodontics and for which types of appliances.

## 2. Materials and Methods

An anonymous questionnaire available in two languages, English and Italian, was diffused between patients of different dental practices and from different countries electronically; 120 answers were considered. The survey was composed of questions made originally for this study using a Google form (Google LLC., 1600 Amphitheatre Parkway, Mountain View, CA, USA) and proposed to patients. All collected data were anonymous; all participants provided informed consent and accepted the privacy policy for the protection of personal data before completing the survey. The questionnaire was administered by an online form service (Google Form service, Google LLC., 1600 Amphitheater Parkway, Mountain View, CA, USA). There were no reminders sent to patients, to let them feel free to answer. It was specified that the purpose of the questionnaire was to find ways for clinicians to improve their methods of curing patients [16]. It was ascertained that each patient provided one answer by checking the timing and for different kinds of response. Every answer that did not respect those parameters was excluded from the study. Six answers were not included. Three of these had the same timing with identical choices and the other three questionnaires were returned without responses. All these were excluded from data analysis before performing the statistical analysis. The patients were asked to complete the questionnaire without any possible compensation or benefit in return. The questionnaire was compiled specifically for this study and, due to the contingency of the COVID-19 pandemic waves, pre-testing was not a viable option. Moreover, they were taken in consideration of the limits of an anonymous questionnaire, in particular, the possibility of misunderstanding the questions and, consequently, giving wrong answers.

The questions concerned:

- Demographic data;
- Level of education;
- If they had an orthodontic therapy in the last two years and if it is finished;
- The kind of treatment, if fixed or clear aligners;
- The type of DI they had;
- The times of DI taking;
- The level of annoyance determined by the two types of techniques;
- The grade of satisfaction determined by the orthodontic therapy;
- The perception of the costs.

## 3. Statistical Analysis

We performed chi-square and Fisher Test for dichotomous variables; age was tested for normality, and Kruskal–Wallis, for multiple groups, was performed to analyze aligner therapies and age groups. Clear aligners were approached using the Student t test for independent samples.

## 4. Results

We enrolled 120 responders, whose demographic characteristics are summarized in Table 1.

The choice of impression is correlated with the type of oral device: excluding those who did not take any impression or those who took an impression both ways, those who underwent traditional impression were more prone to use fixed devices, while digital impressions were more correlated with clear aligners (*p* < 0.001 OR: 2.44 IC:1.5–3.9)

Impression does not seem to be correlated with treatment satisfaction or number of impressions necessary to complete treatment, while the cost of treatment was rated higher in those who underwent both impression (median 8.7) followed by those who underwent none (8.5), only digital (7.8) or only traditional (6.9).

**Figure 1:**
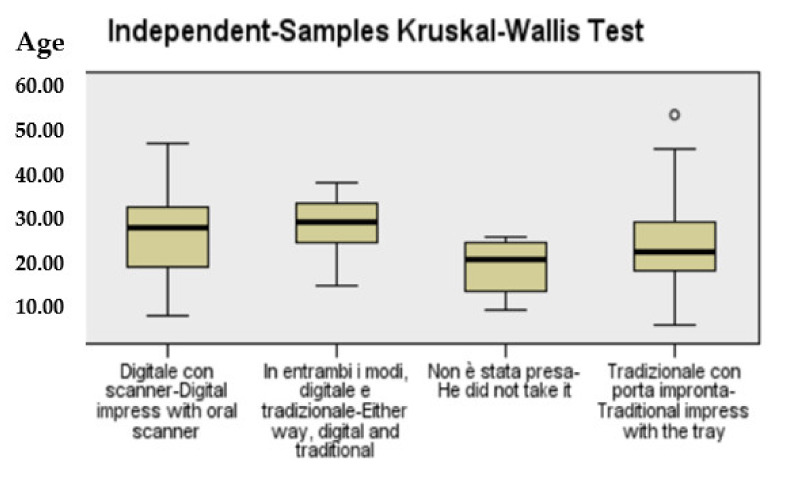
Kruskal-Wallis Test.

We performed the Kruskal–Wallis test for age, for impression and for therapy groups, finding no correlation with the kind of impression. There was a significant correlation between the type of impression and the kind of therapy (*p* < 0.05), while aligners were more used in older patients (34.0 ± 7.2) and fixed therapy in the younger ones (30.1 ± 8.8 (Figure 1).

There were no significant influences on the choice of impressions and orthodontic devices by degree of education, occupational fields or geographical area.

There was no correlation between the kind of impression or device and the probability of completing the treatment (Table 2).

Clear aligners needed more impressions than fixed devices (1.87 ± 0.11 vs. 1.53 ± 0.11 *p* < 0.05), while kind of impression chosen seems not to be relevant (Table 2).

## 5. Discussion

Dental impression taking is one of the most common dental procedures. It is the first step in prosthetic and orthodontic cures, and, often, it is an indispensable step to develop a correct therapeutic plan. In terms of patients’ acceptability and comfort, often the traditional imprint is poorly tolerated. It is common, in fact, that patients complain of a sense of suffocation and retching, sometimes refusing to submit to the impression taking [17].

The review by Ciucciù et al. shows that the digital impression is very effective in reducing anxiety and nausea; it can be considered more comfortable for patients than a conventional impression technique [5]. Furthermore, for the patients, the digital impression reduces the annoyances [17,18,19]; instead, for the dentist, this new technology helps saving time and improves job’s quality [14,20]. The study conducted by Lee et al. reported that the use of intraoral scanners was more efficient and took less time than traditional methods for implant impressions, especially considering inexperienced operators [18]. Considering the timing of scanning, in recent literature, it was affirmed that there is a significant difference between the devices that are now used by clinicians. Yuzbasioglu et al. [17] reported that comparing time scanning, using Trios and iTero, spent by operators at the beginning of the learning curve and by clinicians with an important experience, it was evident that Trios had a shorter average of time scanning. The time scanning and the accuracy depend also on different other aspects [21,22], as the grade of fluidity of the movements of the clinician and his precision during the procedure; in particular, the capability to maintain a constant distance from the teeth can influence these parameters. It is important to affirm that other factors like saliva, blood, clinical expertise influence the results obtained by this technology. However, the high costs and the need to learn how to use it, determined a lower spread of this new kind of devices [23,24].

Digital impression technology involves the use of an intraoral scanner. There are different types of scanners on the market. The first chair-side CAD/CAM system was CEREC (Sirona Dental Systems, Bensheim, Germany), developed over 30 years ago at the University of Zurich. Subsequently, various intraoral scanning systems were developed, such as the iTero (Align Technology, San Jose, CA, USA), the True Definition (3M ESPE, St. Paul, MN, USA), the PlanScan (Planmeca/E4D Technologies, Richardson, TX, USA), the CS 3500 (Carestream Health, Rochester, NY, USA), the TRIOS (3Shape A/S, Copenhagen, Denmark) and CEREC AC Omnicam (Sirona Dental Systems, Bensheim, Germany).

Despite the fact that nowadays dentistry is increasingly digitized and avant-garde, still the use of DI is not widespread and many dentists are still reluctant to replace the conventional impression technique with the new digital technology. The reasons for this are the high cost of the device and the need to learn how to use it, the operator must acquire a certain manual ability because the tongue, the saliva and hypertonic frenula can hinder impression taking [25]. For the traditional imprint, the difficulties are principally related to anatomical conformations of the oral cavity (like tongue, frenula), susceptibility to vomiting and feeling of suffocation. Instead, in DI the main difficulties are flexible oral mucosa and smooth surface texture covered with saliva, conditions that are mainly found in partially or totally edentulous patients [26,27]. However, many dentists mistakenly think that the learning curve of the intraoral scanner is very long and complex and that image capture takes too long time. According to Lee et al., dental students and older clinicians have a different perception of traditional and digital impression. It emerged that the ability to take a traditional impressions depends on the operator’s experience, in fact it was more difficult to perform for the student group than the clinician group. In contrast, the difficult to take DIs was the same in both the group. Student preferred digital impression and clinicians traditional impression [28]. So, dental students revealed to be more open-minded about digital innovation. Ivett Roth et al. has shown that there is a correlation between scan time, number of images and training: the scanning time has decreased as the practice increases. The average scanning time of the first impression was 15 min 28 s and the difference between the first and the last procedure was 7 min 41 s [29]. So, even if it is necessary a learning curve to use intra oral scanner, it is possible to improve the ability to take impression saving time and increasing in the scanning speed of digital impression taking.

Our study underlines that although technology is now present in many aspects of orthodontics, not all dentists use digital impressions. The DI is widespread mainly in the field of invisible orthodontics, that is, among the orthodontic branches, the one that now needs the scanner and digital impressions, considering the companies that produce aligners promote the use of DI, unlike dental technicians that do not have laboratories that are able to acquire digital data [30]. The use of DI is not only beneficial for the patient but also for the operator, in fact, it allows them to realize faster and more standardized procedures. Moreover, the possibility of pre-visualizing the outcome of the treatment and showing it to the patient makes the digital impression also very useful as a marketing strategy [31].

Moreover, it is evident that aligners are more requested by adult patients for aesthetic reasons [4]. In this way, the use of the intraoral scanner can be an important tool in adult orthodontic treatments. In fact, the patients undergoing an orthodontic therapy with aligners required a higher number of impressions. It seems that the satisfaction of the therapy does not depend on the kind of the impression taken.

One of the main problems with the digital impression is the high cost which, as the results of this study highlight, determines a major expense for both the clinician and the patient. However, different scientific articles have demonstrated the efficacy of the intraoral scanner in other branches of dentistry, as well: according to Takeuchi et al., the direct and indirect restorations produced using a DI exhibited an acceptable marginal adaptation [32]. The different uses of the scanner can cover the costs that this technology has for dentists. In the future, it is desirable that affordable costs can make the digital flow more used for pediatric orthodontics. Often, it is precisely the taking of the impression that can be a great annoyance for many children and could be the cause of not starting an orthodontic therapy. In fact, as it arises from the literature, digital impressions are more tolerated by pediatric patients [6]. However, the results of this study highlighted how the type of impression used is not linked to age but much more to the type of device adopted by clinicians. Aligners determine a higher incidence of the use of DI. It is evident that in the orthodontic field, the digital intraoral scanner is necessary to produce customized devices such as aligners, but also to fix orthodontic instruments, such as a 3D Printed Orthodontic Distalizer that is very useful in the second dental class [33]. In general, it is evident that virtual images and digital techniques are changing the clinical approach and viewpoints [34].

Limitations of the study: All the limits of an anonymous questionnaire were taken into consideration. The questionnaire was compiled specifically for this study and, because of the contingency of the COVID-19 pandemic waves, pre-testing was not a viable option. One of the problems of the study is the possibility of misunderstanding the questions and, consequently, giving wrong answers. Moreover, another limit of this study is the major prevalence of females over males in the sample. Another limitation of the study is the relative small sample size, which limits the study’s power to adequately assess the several confounding factors involved, such as the geographical area or the occupational field, and their potential interactions. Moreover, the distribution of the cases within the various subgroups is not even, resulting in several subgroups with very limited representation. Therefore, the current findings regarding the contribution of these factors to the outcomes need to be treated with caution. Furthermore, most of the sample is composed of patients with a high level of education.

## 6. Conclusions

It is right to say that, despite the fact that technology has long since entered into dentistry, and even more so in the last two years, the digital impression has not replaced the traditional one. This could be caused by several factors: first, the high cost of the intraoral scanner and, second, the learning curve required to be capable of using it, in particular for elder dentists. This study confirms that, in the orthodontic field, this new way takeoff taking impressions is mainly used in aligner therapy, considering the advantages of a digital workflow for dental technicians and companies producing these devices. More studies are certainly needed to compare the accuracy of traditional and digital impression so that the latter can also become much more widespread in the field of fixed orthodontics and prosthodontics.

## Figures and Tables

**Table 1:** Demographic data expressed as absolute number (percentage).

Demographic Data	
Age	32.2 ± 8.2
	Education
Middle High or Lower	6 (5.0%)
High School	37 (30.8%)
College or Higher	77 (64.2%)
	Gender
Female	104 (86.7%)
Male	15 (12.5%)
Non-declared	1 (0.8%)
	Employment
Salaried Worker	54 (45.0%)
Public Sector Employee	24 (20.2%)
Student	17 (14.2%)
Freelance	11 (9.2%)
Unemployed	8 (7.5%)
Homemaker	5 (4.2%)
	Geographical area
Europe	79 (65.8%)
North America	26 (21.6%)
Asia	10 (8.3%)
Central America	2 (1.7%)
Africa	2 (1.7%)
South America	1 (0.8%)

**Table 2:** Comparison between fixed devices and aligners.

	**Fixed**	**Aligner**	
Traditional	33	14	*p* < 0.01
Digital	13	35	
Both	5	16	
None	4	0	
Completed treatment			
Yes	14	12	N.S
No	35	42	
Number of impressions needed	1.53 ± 0.11	1.87 ± 0.11	*p* < 0.05
Age	30.1 ± 8.8	34.0 ± 7.2	*p* < 0.05

## Data Availability

The data that support the findings of this study are available from the corresponding author (S.S.) upon reasonable request.
